# Investigation of the Mechanism of Total Flavonoids From Chuju in Mitigating Cerebral Ischemia–Reperfusion Injury in Rats Using Untargeted Metabolomics

**DOI:** 10.1002/fsn3.72216

**Published:** 2026-08-27

**Authors:** Guanglei Zhang, Zongmeng Zhang, Cong Wang, Gaocheng Shi, Mengqi Zhao, Xianxi Wu, Xiaoyong Yuan, Hao Yu

**Affiliations:** ^1^ College of Traditional Chinese Medicine Bozhou University Bozhou China; ^2^ School of Food Science and Engineering Anhui Science and Technology University Chuzhou China; ^3^ Center of Molecular Metabolism Nanjing University of Science and Technology Nanjing China; ^4^ School of Pharmaceutical Science Anhui University of Chinese Medicine Hefei China

**Keywords:** cerebral ischemia–reperfusion injury, mTOR/4EBP1/eIF4E signaling pathway, non‐targeted metabolomics, total flavonoids of Chuju

## Abstract

Chuju ( *Dendranthema morifolium* (Ramat.) Tzvel. cv.), a traditional Chinese medicinal herb with both medicinal and edible properties. It is widely used in tea beverages and functional foods due to its rich flavonoid and polysaccharide content. Our previous studies demonstrated that the total flavonoids of Chuju (TFCJ) alleviate cerebral ischemia–reperfusion injury (CIRI) in rats. However, the potential metabolic pathways regulated by TFCJ remain unclear, and its therapeutic targets require further investigation. This study aims to elucidate the neuroprotective mechanisms of TFCJ. LC–MS analysis identified 42 compounds within TFCJ. Animal experiments showed that TFCJ significantly reduced cerebral infarct volume, elevated SOD and GSH‐Px levels, and decreased MDA levels in the brain. Non‐targeted metabolomic analysis suggested that TFCJ exerts its protective effects by modulating the tricarboxylic acid (TCA) cycle, glycolysis, pentose phosphate pathway (PPP), and aspartate–glutamate metabolism. Pathway analysis revealed that the 4EBP1/eIF4E signaling pathway may mediate the protective effects of TFCJ. In conclusion, this study analyzed the main components of TFCJ and provided evidence suggesting that the 4EBP1/eIF4E signaling pathway might serve as a potential mediator of its neuroprotective effects against CIRI. These findings provide a theoretical basis for the application of TFCJ as both a functional food and a medicinal agent.

## Graphical abstract

Total flavonoids of Chuju (TFCJ) protect against cerebral ischemia–reperfusion injury (CIRI) in rats by reducing infarct volume, alleviating oxidative stress, and regulating metabolic pathways including the TCA cycle, glycolysis, pentose phosphate pathway (PPP), and aspartate–glutamate metabolism. The 4EBP1/eIF4E signaling pathway serves as a crucial mediator underlying the neuroprotective effects of TFCJ.

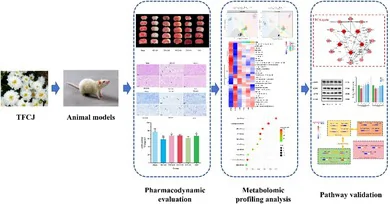

## 1 Introduction

Stroke represents a leading global cause of disability and mortality, exerting a substantial economic burden on healthcare systems and affected families. Clinically, stroke is defined as an acute cerebrovascular event characterized by ischemic injury and subsequent damage to brain tissue resulting from impaired blood flow to specific cerebral regions (Hu et al. 2017). Stroke encompasses two primary subtypes: ischemic stroke (IS) and hemorrhagic stroke (HS), with IS constituting approximately 75% to 85% of all cerebrovascular incidents (Andersen et al. 2009). IS is associated with elevated rates of mortality, morbidity, and disability (Lo and Fukuda 2025), with more than 70% of survivors experiencing a loss of independent living capacity (Powers 2020), thereby significantly diminishing their quality of life. Data from the Global Burden of Disease study in 2019 indicate that China reported 3.49 million new stroke cases, accompanied by a concerning trend toward younger affected populations (Ma et al. 2021).

Cerebral ischemia, caused by a transient or persistent reduction in blood flow, deprives the brain of glucose and oxygen, resulting in infarcted regions. Neurons in the severely hypoperfused infarct core suffer irreversible damage, while those in the surrounding ischemic penumbra remain metabolically active for some time (Yang et al. 2025). Depending on the treatment administered, cells in the penumbra may either progress to death or recover (Puig et al. 2018). Intravenous thrombolysis and thrombectomy are currently the primary clinical treatments for IS. However, restoring blood flow to ischemic brain tissue does not always reverse reversible injuries (Gerschenfeld et al. 2017). Instead, it may exacerbate irreversible damage, such as neuronal apoptosis and necrosis, a phenomenon known as cerebral ischemia–reperfusion injury (CIRI) (Q. Zhang et al. 2022). CIRI is a critical pathological process in IS, primarily associated with ischemia and hypoxia, as well as involving complex pathological mechanisms and multiple cell death pathways (Amani et al. 2019).

Chuju (*Dendranthema morifolium (Ramat.) Tzvel. cv*.), an endemic medicinal and edible herb cultivated in Chuzhou, Anhui Province, is ranked first among China's “Five Famous Medicinal Chrysanthemums.” Renowned for its abundant flavonoids, polysaccharides, and essential oils (Yin et al. 2025). Chuju is widely utilized in functional products such as Chuju liquor and Chuju tea, as well as for essential oil extraction. In the 2025 edition of the Chinese Pharmacopeia, Chuju is identified as one of the primary chrysanthemum varieties. Traditionally, as a medicinal chrysanthemum, Chuju is used to reduce liver‐yang and treat symptoms such as dizziness caused by hyperactivity of liver‐yang. Modern studies have demonstrated that Chuju exhibits pharmacological activities such as anti‐myocardial ischemia (Wang, Chen, et al. 2021), hypoglycemic (Yin et al. 2025), anti‐inflammatory (Han et al. 2015), and antioxidant (Zhan et al. 2022) effects, with flavonoids identified as its principal bioactive components. Our previous research confirmed that the total flavonoids of Chuju (TFCJ) effectively protect rats against CIRI by inhibiting oxidative stress and apoptosis (Wang, Chen, et al. 2021).

Due to its multiple components, TFCJ exhibits characteristics of acting on multiple targets and pathways. In addition to its antioxidant and anti‐inflammatory effects, its actions on key nodes of cellular metabolism and its protective effects on the nervous system through the regulation of energy metabolism responses have yet to be fully elucidated. In this study, a rat model of CIRI was established to evaluate the protective effects of TFCJ. Non‐targeted metabolomics was used to detect changes in brain tissue metabolites across different experimental groups, and systematic metabolomic analyses were conducted to predict and validate potential metabolic pathways and targets. This investigation enhances the understanding of TFCJ's pharmacological effects, chemical composition, and mechanisms of action, potentially uncovering novel therapeutic targets and facilitating the development of new drugs.

## 2 Materials and Methods

### 2.1 Materials

Chuju was provided by Anhui Jutai Chuju Grass‐Rooted Technology Co. Ltd. and authenticated as genuine by Director Meng Xiangsong, a chief pharmacist at the College of Traditional Chinese Medicine, Bozhou University. Xuesaitong, used as a positive control, was purchased from KPC Xuesaitong Pharmaceutical Co. Ltd. Formic acid and acetonitrile (HPLC grade) were procured from Fisher Scientific (MA, USA). The 2,3,5‐Triphenyltetrazolium chloride (TTC) reagent was obtained from Sinopharm Chemical Reagent Co. Ltd., while Hematoxylin and Eosin (HE) and Nissl staining kits were sourced from Beijing Solarbio Science & Technology Co. Ltd. (Beijing, China). Superoxide dismutase (SOD), glutathione peroxidase (GSH‐Px), and malondialdehyde (MDA) assay kits were acquired from the Nanjing Jiancheng Bioengineering Institute (Nanjing, China). The following antibodies were utilized: p‐4EBP1 (Thr37) Polyclonal Antibody (YP‐Ab‐03507), 4EBP1 Polyclonal Antibody (YP‐Ab‐03669), and eIF4E Rabbit mAb (YP‐Ab‐17,916), all procured from UpingBio Technology Co. Ltd. (Hangzhou, China). The β‐actin antibody (catalog number GB11001) was obtained from Wuhan Servicebio Co. Ltd. (Wuhan, China).

### 2.2 Preparation of TFCJ

The dried Chuju powder underwent ultrasonic extraction with 70% ethanol at a material‐to‐liquid ratio of 1:20. The extraction was performed at 60°C for 60 min. The filtrate was then collected, concentrated, and purified using D101 macroporous adsorption resin (Sinopharm Chemical Reagent Co. Ltd., Shanghai, China). Rutin was used as the reference standard to determine the TFCJ via ultraviolet spectrophotometry, yielding a value of 85.58% (Total flavonoid content expressed as rutin equivalents). The chemical profile of TFCJ was further analyzed and identified through liquid chromatography–mass spectrometry (LC–MS) (Sinopharm Chemical Reagent Co. Ltd., Shanghai, China) to facilitate subsequent experiments.

### 2.3 Animals

This study utilized SPF‐grade male Sprague–Dawley (SD) rats, which were purchased from the Experimental Animal Center of Hangzhou Medical College (Production License Number: SCXK (Zhe) 2019‐0002; Qualification Certificate Number: 20230822Aazz0100000530). Before modeling, the rats underwent a one‐week adaptive feeding period, during which they were allowed free access to food, water, and activity in a well‐ventilated environment with a 12‐h light/dark cycle. All animal housing and experimental procedures were approved by the Animal Ethics Committee of Anhui Science and Technology University (Chuzhou, China; approval number: AK2024044) and were carried out in compliance with the guidelines set forth by the European Community (EEC Directive of 1986; 86/609/EEC).

### 2.4 Animal Grouping and Drug Administration

Male SD rats were weighed and numbered. A random sequence was generated using a random number table, and the animals were allocated into six groups according to this sequence. There was no significant difference in mean body weight among the groups (*n* = 9 per group): (1) Sham operation group (Sham); (2) middle cerebral artery occlusion (MCAO) group: (I/*R* + saline); (3) TFCJ low‐dose (TFCJ‐L) group: (I/*R* + 25 mg/kg TFCJ); (4) TFCJ medium‐dose (TFCJ‐M) group: (I/*R* + 50 mg/kg TFCJ); (5) TFCJ high‐dose group (TFCJ‐H): (I/*R* + 100 mg/kg TFCJ); (6) Positive control group (XST): (I/*R* + 25 mg/kg Xuesaitong). TFCJ solutions were prepared at concentrations of 2.5 mg/mL, 5.0 mg/mL, and 10.0 mg/mL for subsequent administration. All groups were administered the respective treatments via intragastric gavage once daily for seven consecutive days. The volume of gavage was maintained at 1 mL/100 g body weight. One hour after the final administration, the rats underwent modeling procedures.

### 2.5 Preparation of the Rat CIRI Model

In order to minimize mortality, the rats were fasted for 12 h prior to the surgical procedure but had unrestricted access to water. Under 4% isoflurane anesthesia, the right sternocleidomastoid and sternohyoid muscles were separated to expose the common carotid artery (CCA). A monofilament suture was carefully inserted into the internal carotid artery (ICA) via the external carotid artery (ECA) and CCA to occlude the origin of the middle cerebral artery (MCA), following the procedure described in previous studies (Wu et al. 2020). After 1 h of ischemia, the filament was removed to restore blood flow, initiating reperfusion, which lasted for 24 h. In the sham group, the CCA was exposed without suture insertion or disruption of blood flow. Neurological function scores were assessed 24 h after modeling. The success rate of the MCAO surgery was approximately 90%. Animals that experienced severe bleeding during the operation or those in each treatment group with neurological deficit scores of 1 or 5 after modeling were excluded and promptly replaced. A score of 1 indicates no obvious neurological impairment, while a score of 5 indicates that the animal is unconscious, unresponsive, and unable to walk independently.

### 2.6 Neurological Function Assessment

Twenty‐four hours after reperfusion, neurological function was assessed by researchers blinded to the group allocations (Joya et al. 2021), the scoring criteria are provided in Supplementary Table S1. The animals were euthanized after the evaluation of neurological function, and brain tissues were harvested for further experiments. Neurological function assessments were performed by investigators blinded to group assignment.

### 2.7 Infarct Volume Measurement

Brain tissues were collected and placed at −20°C for approximately 30 min until slightly hardened. The brains were then sectioned into 2‐mm‐thick coronal slices. The slices were incubated in a 3% TTC solution at 37°C in darkness for 15 min, with the slices flipped once at approximately 7 min to ensure uniform staining. After staining, the slices were rinsed with normal saline and photographed. Infarct volumes were measured using ImageJ software. Infarct volume (%) was calculated as (sum of white volume across all sections/total volume of all sections) × 100%.

### 2.8 Histopathological Examination

Brain tissues from each group were fixed in 10% formalin and sectioned into 5‐μm slices. HE staining was performed, followed by deparaffinization in xylene, rehydration through a graded ethanol series, and subsequent staining as follows: as previously described (Z. Zhang et al. 2018). The samples were then observed under a light microscope. Key features, including ischemic tissue damage and necrotic neuronal characteristics, were analyzed under the microscope.

Paraffin sections were deparaffinized using xylene, dehydrated through a graded ethanol series, and stained with Nissl stain. The stained sections were then observed under a light microscope to assess neuronal damage in brain tissue. Histopathological assessments were performed by investigators blinded to group assignment.

### 2.9 Antioxidant Activity Assay

Brain tissues were accurately weighed and homogenized with pre‐cooled normal saline at a 1:9 (w/v) ratio. Following a 10‐min centrifugation at 4000 rpm, the supernatant was collected from the homogenate for analysis. Following the reagent kit instructions, the activities of SOD, GSH‐Px, and the MDA content in brain tissues were measured.

### 2.10 LC–MS Metabolomics Analysis

#### 2.10.1 Sample Preparation

Approximately 30 ± 5 mg of damaged brain tissue from the affected side was weighed and placed into a 2 mL tube. To each sample, 450 μL of pre‐chilled MeOH: H_2_O (4:1, v/v) was added, along with ceramic beads. The solvent volume was adjusted based on the tissue weight. The sample was homogenized using a homogenizer and left at −20°C for 1 h. Subsequently, the mixture underwent centrifugation at 13,000 rpm for 10 min at 4°C. The supernatant was collected and dried under a nitrogen (N_2_) stream. The residue was re‐dissolved in an equal volume of MeOH: H_2_O (1:1) and centrifuged again. The supernatant was transferred to a UHPLC sample vial for LC–MS analysis. To guarantee the stability and reproducibility of LC–MS measurement data, rigorous quality control procedures were adopted throughout the present study. First, all test samples underwent standardized pretreatment including thawing, centrifugation and supernatant filtration prior to injection to ensure consistent sample preparation conditions. Second, pooled quality control (QC) samples were prepared by mixing equal volumes of all experimental samples, which could comprehensively represent the overall metabolic profile of the cohort and were used to monitor instrument status, system stability and data repeatability.

#### 2.10.2 UHPLC‐Q‐TOF‐MS Analysis

The prepared brain tissue samples were analyzed using an ultra‐high‐performance liquid chromatography quadrupole time‐of‐flight mass spectrometer (UHPLC‐Q‐TOF‐MS; AB SCIEX Triple TOF 5600+, USA). In terms of injection sequence arrangement, three QC samples were injected in advance before analyzing experimental samples to equilibrate the instrument and stabilize the chromatographic system, eliminating initial signal drift. During data acquisition, one QC sample was inserted every six test samples to continuously monitor instrument response, retention time, and baseline stability.

#### 2.10.3 Chromatographic Conditions

A temperature of 40°C was maintained for the column. A gradient elution program, as described in Supplementary Table 2, was used for the mobile phase, which comprised 0.1% formic acid in water (solvent A) and acetonitrile (solvent B). The system was configured with a flow rate of 0.4 mL/min and an injection volume of 10 μL. The automatic sampler was kept at a constant temperature of 4°C for the duration of the analysis.

#### 2.10.4 Q‐TOF Mass Spectrometry Analysis

Mass spectrometric data were acquired using a hybrid quadrupole time‐of‐flight mass spectrometer (Triple TOF 5600+; AB Sciex, USA). N_2_ served as both the nebulizing and auxiliary gas in the ion source, with both GS1 and GS2 set at 55 psi. The curtain gas was set to 35 psi, and the ion source temperature was 550°C. The spray voltage was 5500 V (+) and −4500 V (−). During the TOF MS‐IDA‐MS/MS acquisition, the TOF MS spectrum was scanned across a mass range of 50 to 1000 m/z, with each spectrum accumulating for 0.10 s. For the product ion scan, the mass range was set between 40 and 1000 m/z, with each spectrum accumulating for 0.05 s. Product ions were scanned using information‐dependent acquisition (IDA) in high‐sensitivity mode. Dynamic background subtraction (DBS) was used to enhance sensitivity for detecting analytes present in low abundance or trace amounts. Every five samples, an external calibration was carried out to guarantee accurate TOF‐MS and TOF‐MS/MS calibration.

#### 2.10.5 Mass Spectrometry Data Processing

Analyst TF 1.8.1 software was used to collect data (developed by SCIEX). Raw *.wiff.scan files were converted to *.mzmL format using ProteoWizard. Data peak processing, including peak alignment, retention time correction, and peak area extraction, was conducted using the R programming language (http://cran.r‐project.org/). The resulting metabolite MS/MS spectra were matched with the HMDB database (Human Metabolome Database) to identify specific metabolites (mass error < 10 ppm). Partial least squares discriminant analysis (PLS‐DA) was performed on the preprocessed data using the “mixOmics” package in R. The variable importance (VIP) values for metabolites were calculated, and metabolites with VIP > 1 were retained for further analysis.

#### 2.10.6 Differential Metabolite Screening and Identification

Differential metabolites were identified using three criteria: VIP > 1 (from PLS‐DA), *p* < 0.05 (Student's *t*‐test), and FDR‐adjusted q < 0.05 (Benjamini‐Hochberg method). Metabolites meeting all three criteria were defined as differential metabolites. Identification was performed by matching with HMDB, METLIN, MassBank, and an in‐house database based on retention time, m/z, fragmentation patterns, and peak area (mass error < 25 ppm). All differential metabolites reported herein correspond to MSI Level 2 putatively annotated metabolites.

#### 2.10.7 Differential Metabolite Enrichment Analysis

Enrichment analysis of differential metabolites was conducted using the MetaboAnalyst 5.0 web platform (https://www.metaboanalyst.ca). Pathway‐metabolite interactions were visualized using Cytoscape software.

#### 2.10.8 Differential Metabolic Pathways

Metabolites were analyzed for their related pathways using the MetScape plugin in Cytoscape and the pathway‐sharing database (http://www.pathwaycommons.org/), incorporating gene and protein interaction data. Pathway analysis of differential metabolites associated with upstream genes and proteins was conducted using the R package CePa, comparing data across different dosage groups. Key common and significant pathways were identified for all groups.

### 2.11 Western Blotting

Western blotting was employed to validate the expression of corresponding marker proteins in rat brain tissues, based on the predicted signaling pathways from untargeted metabolomics. A 100 mg sample of ischemic cortex tissue from each group was homogenized in lysis buffer (Beyotime, Shanghai, China), incubated on ice for cell lysis, and centrifuged at 12,000 rpm for 10 min. Using the BCA method, the protein concentration was measured after collecting the supernatant. After mixing with a sample buffer, proteins were denatured at 100°C for 15 min and then separated by SDS‐PAGE. Following this, the proteins were transferred to a PVDF membrane, the samples were blocked and sequentially incubated with the primary antibody and then the secondary antibody. After washing, the protein blots were developed using chemiluminescence. Band intensity was quantified using ImageJ software.

### 2.12 Statistical Analysis

All experimental results are expressed as the mean ± standard deviation (SD). Data were analyzed using SPSS 24.0 software. The experimental data were first subjected to homogeneity of variance testing. Homogeneity of variances was assessed using Levene's test. For data with homogeneous variances, a one‐way ANOVA followed by Tukey's HSD post hoc test was performed. For data with unequal variances, Welch's ANOVA followed by the Games‐Howell post hoc test was applied, with *p* < 0.05 considered statistically significant.

## 3 Results

### 3.1 Chemical Composition of TFCJ

**FIGURE 1:**
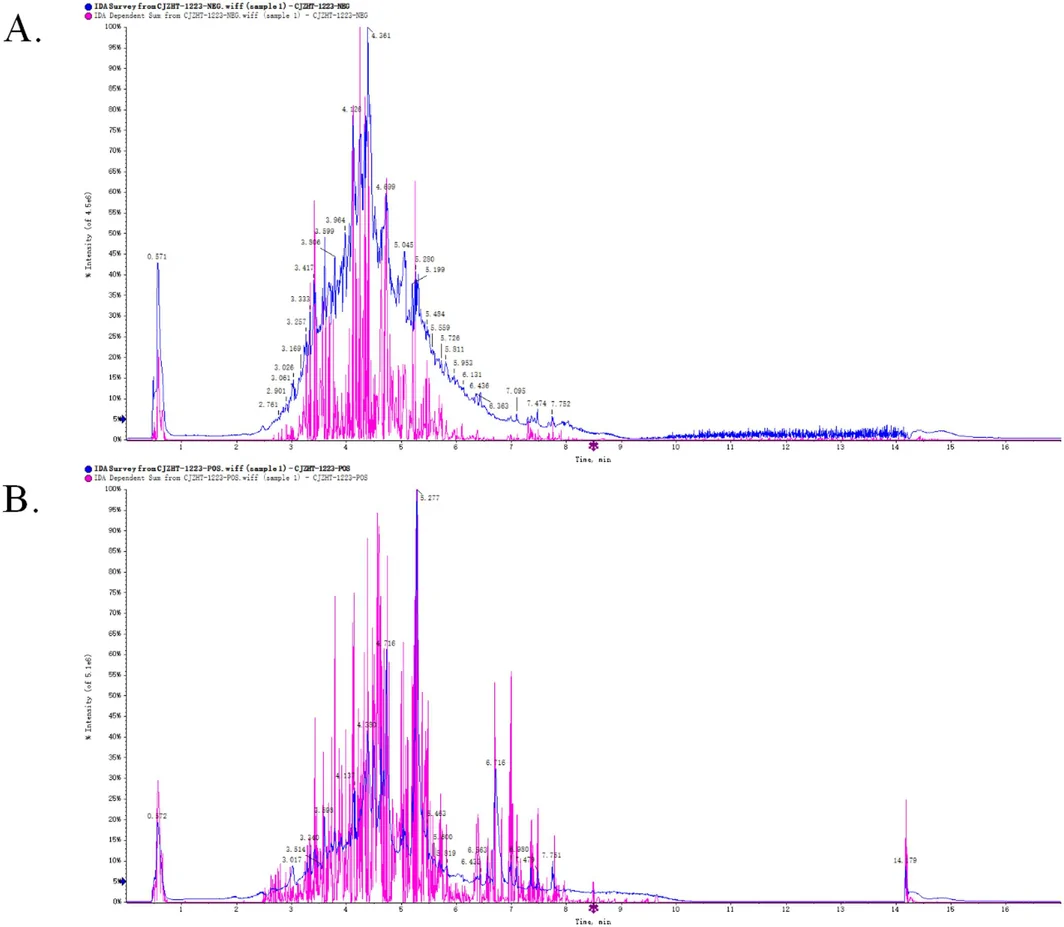
Total ion chromatograms of TFCJ. (A) Negative ion mode (NEG). (B) Positive ion mode (POS).

A total of 42 compounds were identified in the TFCJ. The specific compound information is presented in Table 1. These compounds include apigetrin, luteolin, baicalin, and eriodictyol, among others. Extensive research has demonstrated that these compounds possess notable pharmacological activities, including anti‐inflammatory, antioxidant, and neuroprotective effects (Huang et al. 2021; Lim et al. 2016; Luo et al. 2019; Wang, Ma, et al. 2021). The total ion chromatograms are shown in Figure 1.

**TABLE 1:** Identification results of chemical constituents in TFCJ.

No	RT	mz	MW	calAdduct	Name	MF	Mode
1	3.30	305.07	304.06	[M + H]+	(+/−)‐Taxifolin	C_15_H_12_O_7_	POS
2	3.67	551.1	550.1	[M + H]+	Quercetin 3‐O‐malonylglucoside	C_24_H_22_O_15_	POS
3	3.78	565.15	564.15	[M + H]+	Corymboside	C_26_H_28_O_14_	POS
4	4.04	479.08	478.07	[M + H]+	quercetin 3‐O‐glucuronide	C_21_H_18_O_13_	POS
5	4.08	433.11	432.11	[M + H]+	Isovitexin	C_21_H_20_O_10_	POS
6	4.11	289.07	288.06	[M + H]+	Eriodictyol	C_15_H_12_O_6_	POS
7	4.13	303.05	302.04	[M + H]+	quercetin	C_15_H_10_O_7_	POS
8	4.15	450.11	449.11	[M + H]+	Cyanidin‐3‐O‐glucoside	C_21_H_21_O_11_	POS
9	4.25	551.1	550.1	[M + H]+	Quercetin 3‐(6″‐malonyl‐glucoside)	C_24_H_22_O_15_	POS
10	4.36	609.17	608.17	[M + H]+	Diosmetin‐7‐O‐rutinoside	C_28_H_32_O_15_	POS
11	4.40	433.11	432.11	[M + H]+	Apigetrin	C_21_H_20_O_10_	POS
12	4.44	447.08	446.08	[M + H]+	Baicalin	C_21_H_18_O_11_	POS
13	4.49	464.13	463.12	[M + H]+	Peonidin‐3‐glucoside	C_22_H_23_O_11_	POS
14	4.51	301.07	300.06	[M + H]+	Diosmetin	C_16_H_12_O_6_	POS
15	4.53	317.07	316.06	[M + H]+	Isorhamnetin	C_16_H_12_O_7_	POS
16	4.84	593.18	592.18	[M + H]+	Linarin	C_28_H_32_O_14_	POS
17	5.01	287.05	286.05	[M + H]+	Luteolin	C_15_H_10_O_6_	POS
18	5.05	285.07	284.07	[M + H]+	Acacetin	C_16_H_12_O_5_	POS
19	5.15	461.11	460.1	[M + H]+	Wogonoside	C_22_H_20_O_11_	POS
20	5.66	533.12	532.12	[M + H]+	Malonylglycitin	C_25_H_24_O_13_	POS
21	5.73	361.09	360.08	[M + H]+	2‐(3,4‐dihydroxyphenyl)‐5‐hydroxy‐3,6,7‐trimethoxychromen‐4‐one	C_18_H_16_O_8_	POS
22	6.28	345.09	344.09	[M + H]+	5,7‐Dihydroxy‐3,6,4′‐trimethoxyflavone	C_18_H_16_O_7_	POS
23	6.38	375.1	374.1	[M + H]+	Chrysosplenetin	C_19_H_18_O_8_	POS
24	6.46	375.1	374.1	[M + H]+	Vitexicarpin	C_19_H_18_O_8_	POS
25	2.70	595.15	596.14	[M‐H]−	Quercetin 3‐lathyroside	C_26_H_28_O_16_	NEG
26	3.00	433.11	434.12	[M‐H]−	Eriodictyol 7‐rhamnoside	C_21_H_22_O_10_	NEG
27	3.21	339.08	340.08	[M‐H]−	Esculin	C_15_H_16_O_9_	NEG
28	3.35	639.13	640.13	[M‐H]−	Nelumboside	C_27_H_28_O_18_	NEG
29	3.47	609.16	610.15	[M‐H]−	Luteolin 6‐C‐glucoside 8‐C‐arabinoside	C_27_H_30_O_16_	NEG
30	3.78	563.15	564.15	[M‐H]−	5,7‐dihydroxy‐2‐(4‐hydroxyphenyl)‐8‐[3,4,5‐trihydroxy‐6‐(hydroxymethyl)oxan‐2‐yl]‐6‐(3,4,5‐trihydroxyoxan‐2‐yl)chromen‐4‐one	C_26_H_28_O_14_	NEG
31	3.90	461.08	462.08	[M‐H]−	Kaempferol 3‐glucuronide	C_21_H_18_O_12_	NEG
32	4.03	609.16	610.15	[M‐H]−	Rutin	C_27_H_30_O_16_	NEG
33	4.09	449.12	450.12	[M‐H]−	Miscanthoside	C_21_H_22_O_11_	NEG
34	4.12	477.07	478.07	[M‐H]−	Quercetin glucuronide	C_21_H_18_O_13_	NEG
35	4.16	447.1	448.1	[M‐H]−	Luteolin 7‐glucoside	C_21_H_20_O_11_	NEG
36	4.26	505.11	506.11	[M‐H]−	Quercetin 3‐ (6″‐acetylglucoside)	C_23_H_22_O_13_	NEG
37	4.65	269.05	270.05	[M‐H]−	Apigenin	C_15_H_10_O_5_	NEG
38	5.04	301.04	464.1	[M‐C6H10O5‐H]−	Spiraeoside	C_21_H_20_O_12_	NEG
39	5.21	345.07	346.07	[M‐H]−	5,7,3′,4′‐Tetrahydroxy‐6,8‐dimethoxyflavone	C_17_H_14_O_8_	NEG
40	5.68	597.38	598.4	[M‐H]−	Idoxanthin	C_40_H_54_O_4_	NEG
41	5.73	359.08	360.08	[M‐H]−	Jaceidin	C_18_H_16_O_8_	NEG
42	6.38	373.1	374.1	[M‐H]−	Skullcapflavone II	C_19_H_18_O_8_	NEG

### 3.2 Neuroprotective Effects of TFCJ on CIRI Rats

**FIGURE 2:**
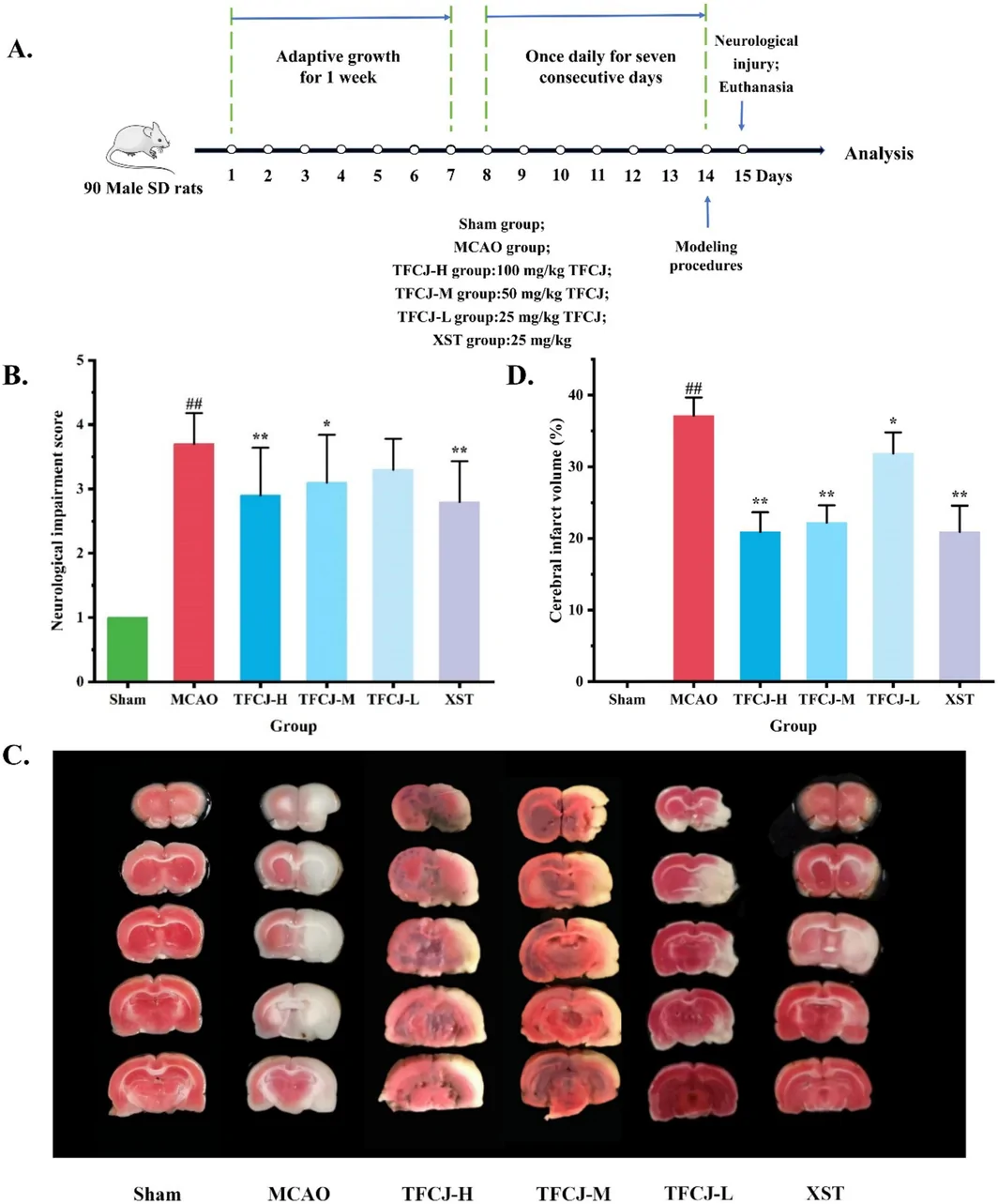
Neuroprotective effects of TFCJ on CIRI rats. (A) Schematic diagram illustrating animal grouping, TFCJ administration, and experimental modeling. (B) Neurological function scores. (C) Schematic diagram of cerebral infarct volume. (D) Quantitative analysis of cerebral infarct volume. ##*p* < 0.01 vs. Sham group; *p < 0.05, **p < 0.01 vs. MCAO group. Data are presented as mean ± SD (*n* = 9).

To investigate the effect of TFCJ on CIRI, a MCAO rat model was established (Figure 2A). The sham group exhibited grade I neurological scores, indicating no symptoms of neurological dysfunction (Figure 2B). In contrast, the MCAO group exhibited significantly elevated neurological scores relative to the sham group (Figure 2B), reflecting marked neurological deficits and confirming the successful induction of the model. Neurological impairment scores in the TFCJ‐H and TFCJ‐M groups were significantly reduced compared to those in the MCAO group (Figure 2B). TTC staining revealed that dehydrogenase activity in normal tissue reacted with TTC, resulting in a red coloration, whereas infarcted ischemic regions with reduced dehydrogenase activity appeared white (Figure 2C). In the MCAO group, distinct white infarct regions were observed in the right cerebral hemisphere, with an infarct volume of 37.14%, representing a significant increase compared to the sham group (Figure 2D). Compared to the MCAO group, infarct volumes in all TFCJ‐treated groups were significantly reduced, demonstrating a dose‐dependent decrease (Figure 2D). These findings indicate that TFCJ protects rats from CIRI.

#### 3.2.1 TFCJ Reduces Tissue Damage and Enhances Antioxidant Capacity in CIRI Rats

**FIGURE 3:**
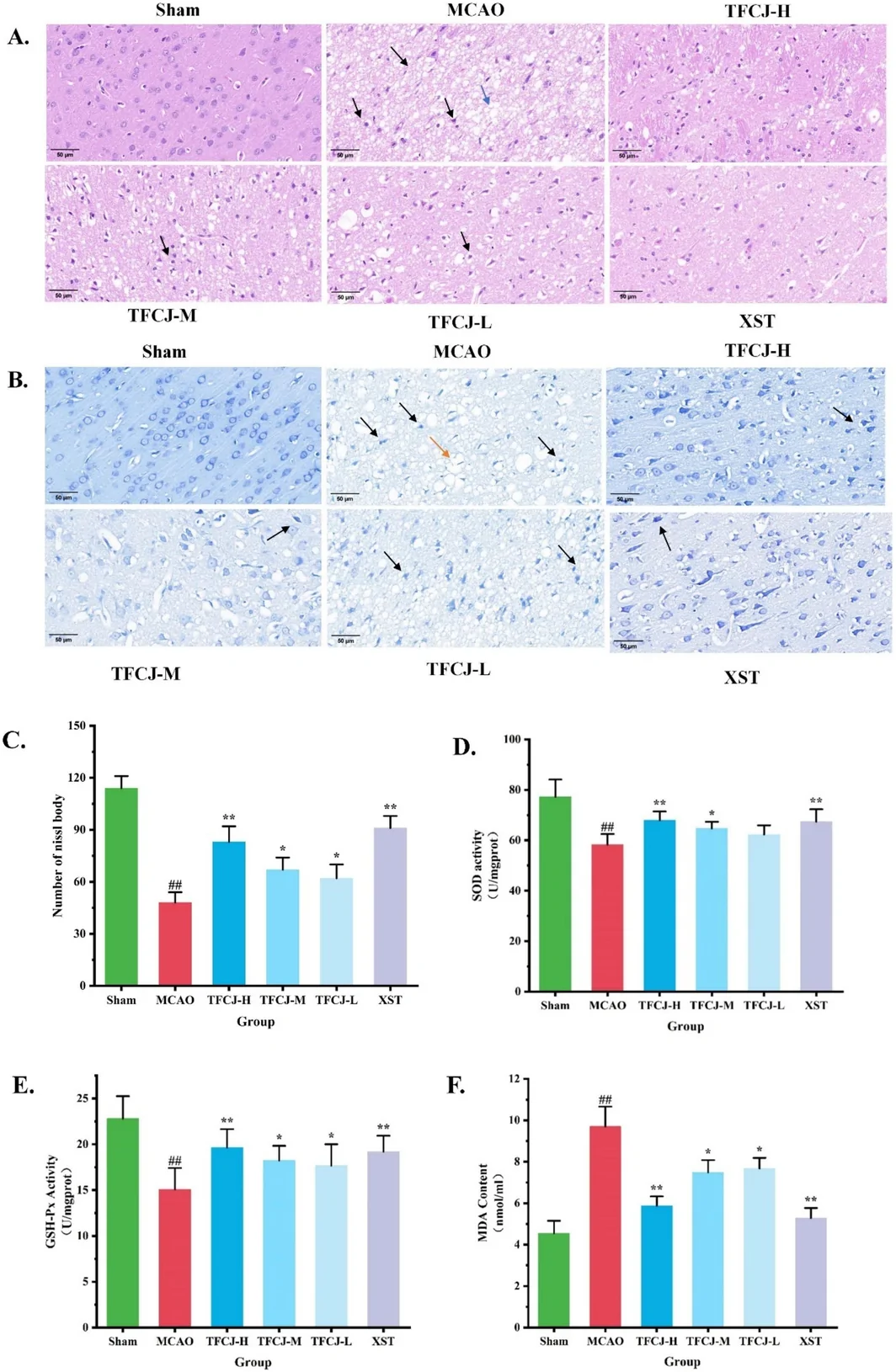
Histopathological and antioxidant capacity evaluation of TFCJ on neuronal damage in CIRI rats. (A) Schematic diagram of HE staining. Scale bar: 50 μm. (B) Schematic diagram of Nissl staining. Scale bar: 50 μm. (C) Quantitative analysis of Nissl body counts. (D) SOD levels in brain tissues across groups. (E) GSH‐Px levels in brain tissues across groups. (F) MDA content in brain tissues across groups. ##*p* < 0.01 vs. Sham group; *p < 0.05, **p < 0.01 vs. MCAO group. Data are presented as mean ± SD (*n* = 9).

The HE staining results for each group are shown in Figure 3A. Neuronal cells with condensed nuclei are indicated by black arrows, while vacuolated regions are marked by blue arrows. The MCAO group exhibited a significant reduction in the number of neurons compared to the Sham group, accompanied by disorganized cell arrangements, nuclear condensation and hyperchromasia, and prominent vacuolation around the cells (Figure 3A). Furthermore, nucleoli appeared indistinct in this group. Treatment with TFCJ markedly ameliorated these histopathological alterations (Figure 3A).

The Nissl staining results are shown in Figure 3B,C. Shrunken neuronal cell bodies are indicated by black arrows, while vacuolated regions are marked by orange arrows (Figure 3B). In the MCAO group, neuronal cell bodies appeared shrunken and irregularly shaped, with disorganized arrangements (Figure 3B). Prominent vacuolation was observed around the neuronal cells, accompanied by nuclear damage. A significant reduction in the number of Nissl bodies was observed, and some neurons exhibited necrosis and dissolution (Figure 3B,C). Compared to the MCAO group, the TFCJ‐treated groups demonstrated improvements in the size and morphology of neuronal cell bodies, more regular arrangements, restoration of Nissl body numbers, and a reduction in vacuolation severity (Figure 3B,C). These results indicate that TFCJ treatment reduces tissue damage in CIRI rats.

To investigate the effects of TFCJ on oxidative stress levels in rat brain tissue, the activities of SOD and GSH‐Px, as well as MDA content, were measured in each group. The experimental results revealed that, compared to the sham group, the MCAO group exhibited significantly reduced SOD activity and GSH‐Px levels, while MDA content was significantly increased (Figure 3D–F). These results indicate that oxidative stress levels in brain tissue markedly increased following CIRI, accompanied by a significant decline in antioxidant capacity. Compared to the MCAO group, SOD and GSH‐Px levels in the TFCJ‐H and TFCJ‐M groups were significantly elevated (Figure 3D,E), while MDA content was significantly decreased (Figure 3F). These findings suggest that TFCJ treatment enhances antioxidant capacity in CIRI rats.

### 3.3 Metabolomics Analysis

#### 3.3.1 PLS‐DA Analysis

**FIGURE 4:**
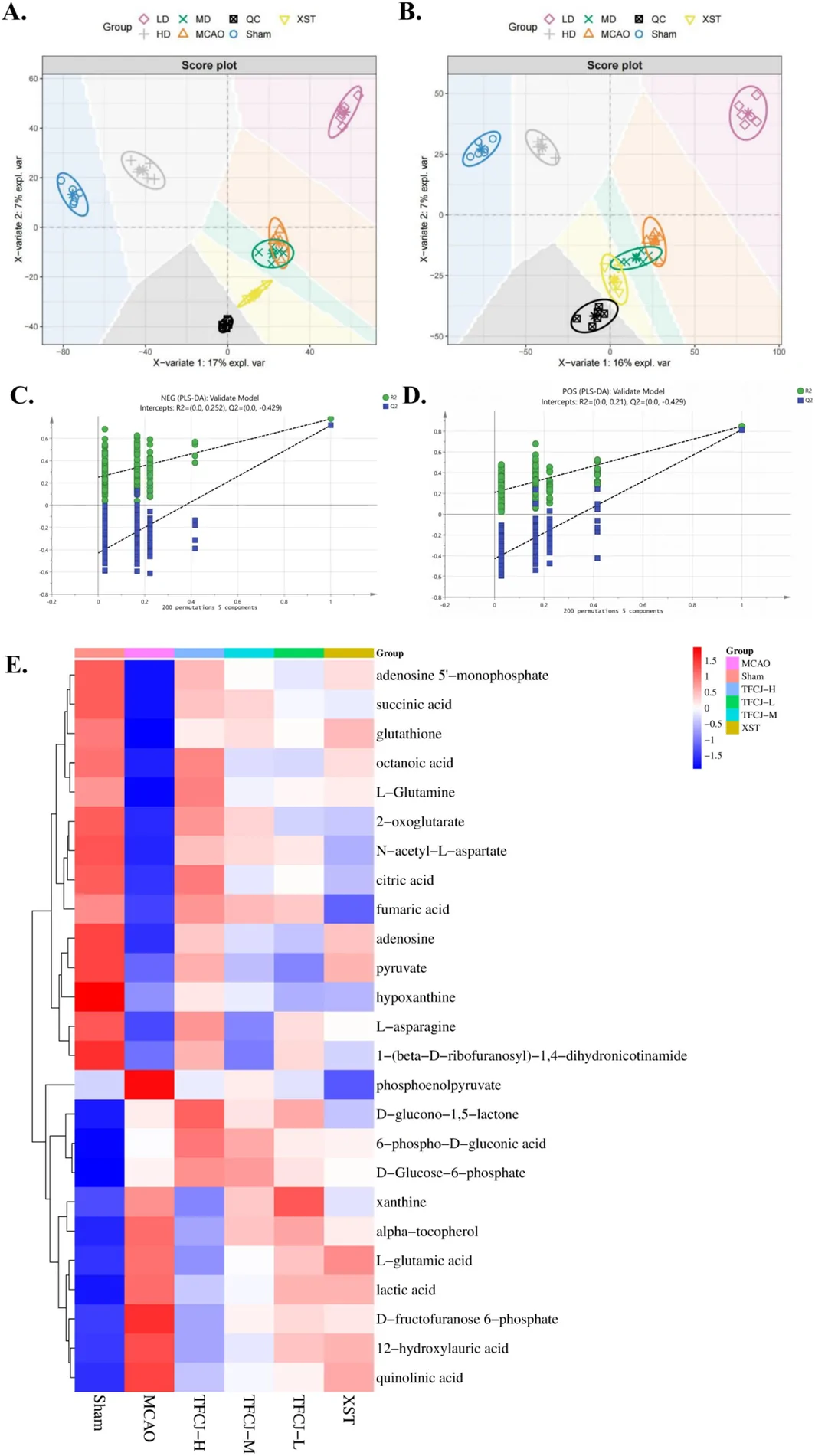
PLS‐DA score plots for each group. (A) NEG and (B) POS. In the figure: LD represents the TFCJ‐L group; MD represents the TFCJ‐M group; HD represents the TFCJ‐H group. (C) Permutation test results (*n* = 200) for the Neg PLS‐DA mode. (D) Permutation test results (*n* = 200) for the Pos PLS‐DA mode. (E) Heatmap of differential metabolites.

PLS‐DA is a supervised classification method specifically designed to analyze complex metabolomics data. By reducing the dimensionality of data and transforming it, PLS‐DA maximizes intergroup differences. Additionally, it enables the identification of differential metabolites between groups by calculating Variable Importance in Projection values. This approach enhances the ability to identify inter‐group differences and provides intuitive visualizations of relationships among samples, including grouping, clustering, and outlier detection. The PLS‐DA scores for each group are shown in Figure 4A,B. The QC group clustered tightly, indicating good stability throughout the experiment. The Sham and Model groups were distinctly separated, indicating significant metabolic perturbations induced by ischemia–reperfusion (Figure 4A,B). Moreover, along the horizontal axis, the TFCJ treatment groups exhibited a progressive distribution in the positive direction (Figure 4A,B), suggesting a potential dose‐dependent trend in metabolic alterations among the experimental animals. Notably, the TFCJ‐H group (HD) clustered nearest to the Sham group (Figure 4A,B), suggesting that the high‐dose treatment resulted in metabolic profiles more closely resembling normal conditions. To further validate the model, permutation tests (*n* = 200) were conducted, yielding R2 values ranging from 0.0 to 0.252 and Q2 values ranging from 0.0 to −0.429. These results indicate the stability and reproducibility of the PLS‐DA model, with no evidence of overfitting, as shown in Figure 4C,D.

#### 3.3.2 Heatmap of Differential Metabolites

Using the screening criteria described in Section 2.10.6 (VIP > 1, *p* < 0.05, and q < 0.05), a total of 25 differential metabolites were identified. These include fumaric acid, citric acid, lactic acid, pyruvate, succinic acid, 2‐oxoglutarate, glutamine, and N‐acetyl‐L‐aspartic acid, among others. The information and changes related to these differential metabolites are presented in Supplementary Table 3. The expression profiles of these differential metabolites are presented in the heatmap (Figure 4E), which provides a visual representation of variations in metabolite levels across the different experimental groups. This highlights patterns of upregulation and downregulation that are critical for understanding the metabolic impact of the treatment.

#### 3.3.3 Enrichment Analysis

**FIGURE 5:**
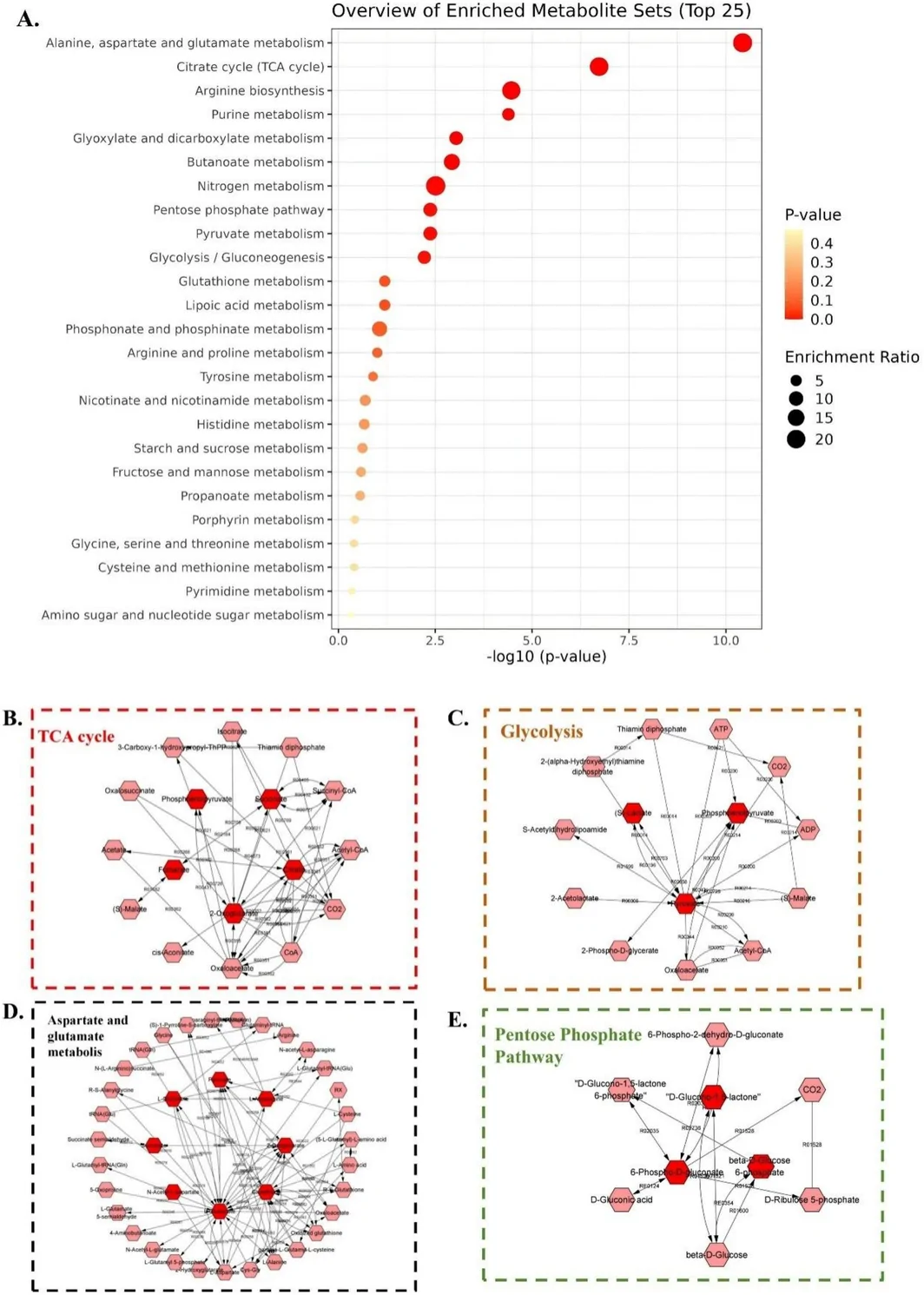
Schematic diagram of major metabolic pathways and their key metabolites (A) Enrichment analysis of differential metabolites. (B) TCA cycle; (C) Glycolysis; (D) Aspartate–glutamate metabolism; (E) Pentose phosphate pathway. The identified differential metabolites are highlighted in red.

Following the methodology outlined in Section 2.10.7, the results of the enrichment analysis are shown in Figure 5A. The analysis revealed that the primary pathways affected involve amino acid metabolism and energy metabolism. Key metabolic pathways include alanine, aspartate, and glutamate metabolism; the TCA cycle; arginine biosynthesis; butanoate metabolism; the pentose phosphate pathway; pyruvate metabolism; glycolysis/gluconeogenesis; and glutathione metabolism (Figure 5B–E). These enriched pathways provide valuable insights into the metabolic alterations caused by CIRI and the therapeutic effects of TFCJ treatment.

### 3.4 TFCJ Exerts a Protective Effect in CIRI Rats Through the 4EBP1/eIF4E Signaling Pathway

**FIGURE 6:**
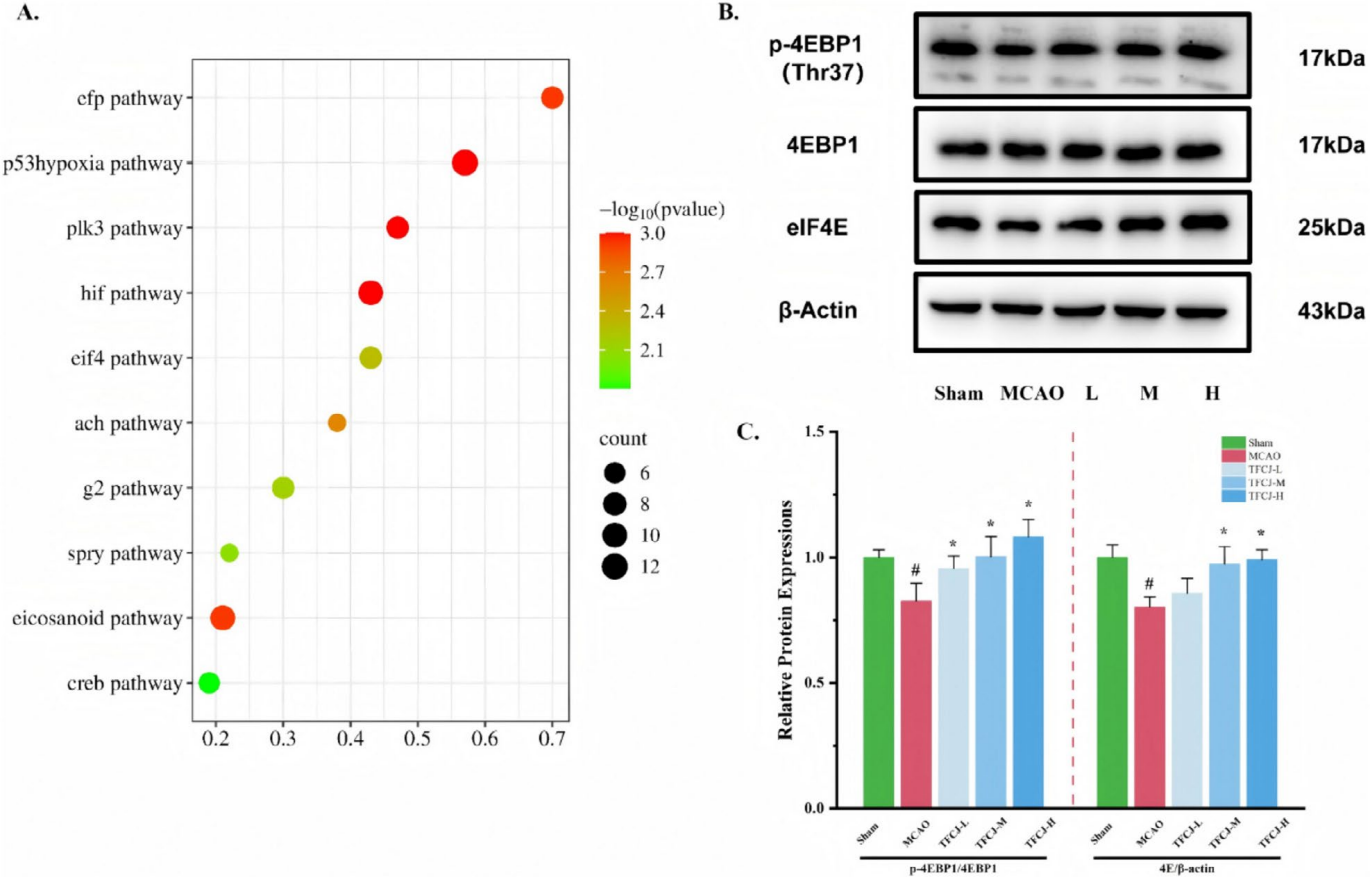
Effect of TFCJ on the 4EBP1/eIF4E signaling pathway. (A) Pathway enrichment analysis plot. (B) Western blot analysis of p‐4EBP1 (Thr37) and eIF4E protein levels. (C) Quantitative analysis of protein expression (*n* = 9). ##*p* < 0.01 vs. Sham group; *p < 0.05, **p < 0.01 vs. MCAO group. Data are presented as mean ± SD.

Metabolites were enriched and analyzed using the MetScape plugin, pathway interaction data from the Pathway Commons database, and the R package. The top 10 core metabolic pathways are shown in Figure 6A. One of the enriched pathways, the eIF4 signaling axis, is a downstream component of the PI3K/Akt/mTOR pathway and involves mTOR‐mediated phosphorylation of 4EBP1, which subsequently releases and activates eIF4E. Our previous study demonstrated that TFCJ exerts neuroprotective effects via the PI3K/Akt/mTOR pathway (Wang, Chen, et al. 2021). Given that mTOR phosphorylates 4EBP1 to release eIF4E and that this axis is known to regulate the translation of metabolic enzymes (including those involved in glycolysis, TCA cycle, and antioxidant defense), we hypothesized that TFCJ may coordinate metabolic recovery via this pathway.

We further examined the protein expression levels of 4EBP1, phosphorylated 4EBP1 (p‐4EBP1), and eIF4E across the experimental groups. Compared to the Sham group, the model group exhibited significantly reduced levels of p‐4EBP1 and eIF4E (Figure 6B,C). However, treatment with TFCJ partially reversed these changes. These results suggest that TFCJ treatment increases the levels of p‐4EBP1 and eIF4E proteins in this pathway. Combined with our previous findings, this suggests that TFCJ may exert a protective effect in CIRI rats potentially through the 4EBP1/eIF4E signaling pathway.

## 4 Discussion

In our study, TFCJ significantly reduced cerebral infarct volume and attenuated ischemia–reperfusion‐induced neuronal loss in MCAO rats. It also enhanced SOD and GSH‐Px activities while reducing MDA levels, suggesting that its neuroprotective effects are partly mediated by antioxidant defense mechanisms.

**FIGURE 7:**
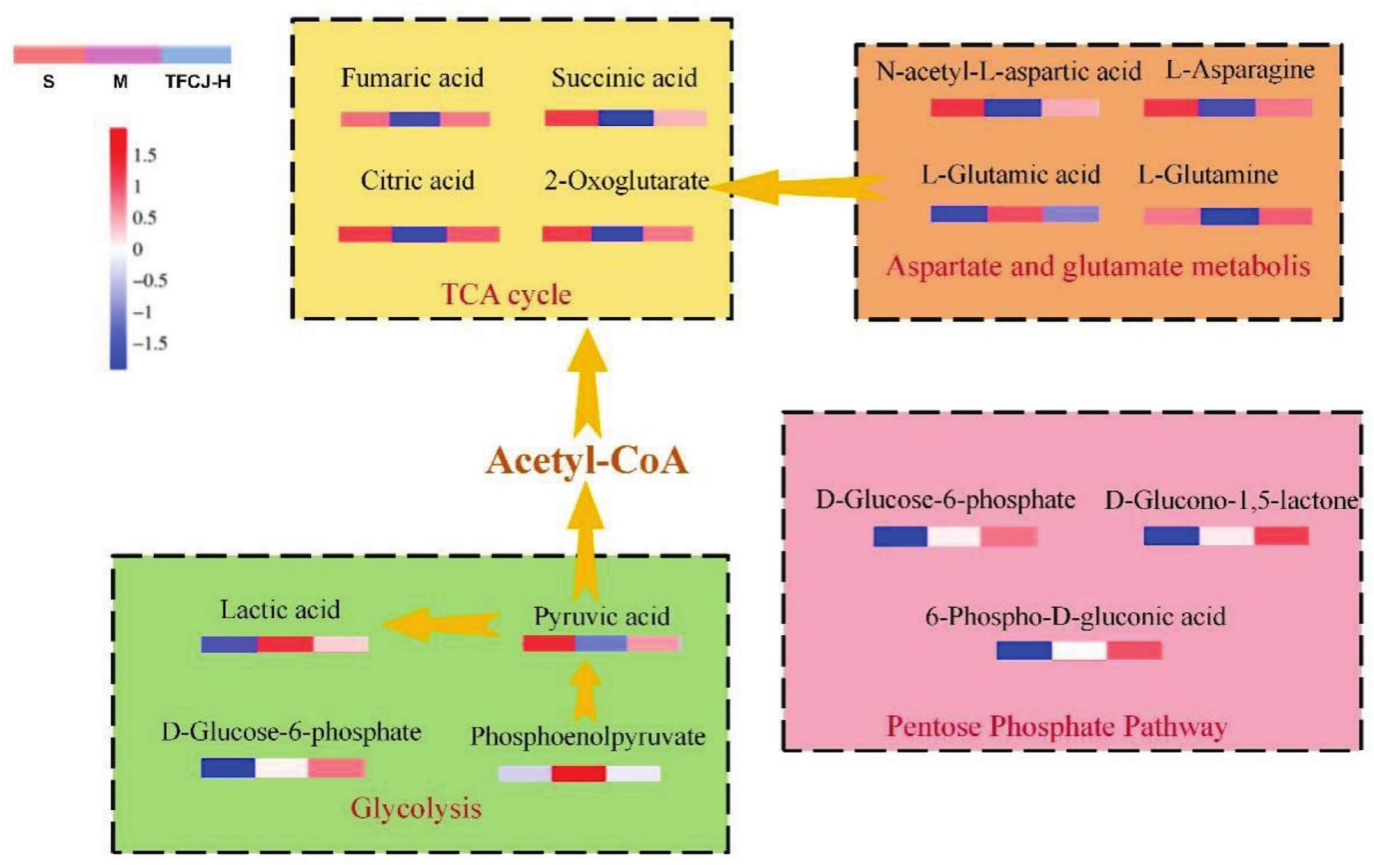
Differential metabolic pathways and changes in metabolites across groups. In the figure: S represents the Sham group; M represents the Model group. The colors of the metabolites indicate different expression levels: Blue: Downregulated expression. Red: Upregulated expression.

Metabolic disturbances contribute to CIRI‐induced neural damage. Further analysis of metabolite enrichment suggests that the protective effects of TFCJ on CIRI involve energy metabolism, amino acid metabolism, and the pentose phosphate pathway (PPP) (Figure 7).

The brain is highly metabolically active, consuming nearly 20% of glucose‐derived energy despite accounting for only 2% of body weight (Wang, Zhou, et al. 2025). The TCA cycle and oxidative phosphorylation generate over 90% of total energy (Bartman et al. 2023). In IS, disruption of TCA cycle flux reduces ATP production and promotes neuronal death (Chen et al. 2019). In MCAO rats, levels of TCA cycle intermediates (citric acid, fumaric acid, succinic acid, α‐ketoglutaric acid) were significantly decreased, while phosphoenolpyruvate and lactate levels were elevated, along with an increased lactate/pyruvate ratio. This pattern suggests compensatory upregulation of glycolysis under hypoxic conditions (Kierans and Taylor 2021). Under ischemic and hypoxic conditions, excessive lactate accumulation exacerbates neuronal damage by promoting calcium influx, anaerobic glycolysis, and acidosis (Wang, Mu, et al. 2025). TFCJ treatment partially reversed these changes, bringing metabolite levels closer to those in the Sham group, indicating improved energy supply and neuroprotection.

The PPP comprises oxidative and non‐oxidative branches. The oxidative branch converts glucose‐6‐phosphate (G6P) to ribose‐5‐phosphate and generates NADPH, which is essential for redox homeostasis and biosynthesis (Chandel 2021). The non‐oxidative branch supplies ribose‐5‐phosphate for nucleotide synthesis. Consistent with previous reports (Vaskova et al. 2023), MCAO rats exhibited elevated levels of G6P, D‐glucono‐1,5‐lactone, and 6‐phospho‐D‐gluconic acid, suggesting PPP upregulation following ischemia–reperfusion. This compensatory response may promote NADPH production and enhance neuronal tolerance to oxidative stress (Cao et al. 2017; Li et al. 2016). TFCJ treatment further increased these metabolites, indicating enhanced PPP activity and improved antioxidant capacity, consistent with the observed reduction in oxidative stress.

Glutamate is a key regulator in the nervous system, but its sustained accumulation leads to excitotoxicity and metabolic dysfunction (Kaplan‐Arabaci et al. 2022). Glutamine serves as both a metabolic fuel and a precursor for glutamate and glutathione. During ischemia–reperfusion, glutamine levels typically decrease while glutamate levels increase (Krzyzanowska et al. 2017). In the MCAO group, glutamine and N‐acetyl‐L‐aspartate (a neuronal damage marker) were reduced, while glutamate levels were elevated, consistent with previous findings (Hasseldam et al. 2024). TFCJ treatment significantly increased glutamine and N‐acetyl‐L‐aspartate levels and decreased glutamate levels. Given that glutamine is a key precursor for glutathione synthesis, these changes further support the role of TFCJ in enhancing antioxidant capacity and neuroprotection.

We previously reported that TFCJ upregulates p‐AKT and p‐mTOR in the PI3K‐Akt–mTOR pathway, reducing apoptosis and oxidative stress (Wang, Chen, et al. 2021). mTORC1 regulates cell growth, protein synthesis, and autophagy, with protein synthesis being particularly vital for neuronal survival and plasticity (Szwed et al. 2021). Following cerebral ischemia, reduced oxygen and glucose levels decrease mTORC1 activity and 4EBP1 phosphorylation (Villa‐Gonzalez et al. 2022). Activation of mTORC1 has been shown to confer neuroprotection after ischemia (Lei et al. 2021; Pan et al. 2022), whereas its inhibition exacerbates neuronal damage (Chi et al. 2021).

In the present study, metabolomic analysis revealed that TFCJ improved metabolic profiles related to the TCA cycle, glycolysis, PPP, and amino acid metabolism. Pathway enrichment analysis identified the eIF4E signaling pathway. Western blotting confirmed that TFCJ reversed CIRI‐induced reductions in p‐4EBP1 and eIF4E protein levels, consistent with activation of the mTORC1/4EBP1/eIF4E axis. However, as the current evidence is correlative, future functional validation using 4EBP1‐specific siRNA or mTOR inhibitors (e.g., rapamycin) is required to establish a causal relationship between pathway activation and the neuroprotective effects of TFCJ.

## 5 Conclusion

In summary, administration of TFCJ significantly alleviated CIRI‐induced neurological damage by improving energy supply and antioxidant capacity. Metabolomics analysis revealed that TFCJ restored the metabolic profile of brain tissues in CIRI rats by upregulating pathways such as the TCA cycle, glycolysis, the pentose phosphate pathway, aspartate metabolism, and glutamate metabolism. Furthermore, our findings suggest that the activation of the 4EBP1/eIF4E signaling by TFCJ might represent a potential mechanism underlying its protection of neural cells in ischemic tissues. These findings offer new understanding into the mechanisms by which TFCJ ameliorates CIRI in rats and offer valuable clues and new perspectives for therapeutic approaches.

## Author Contributions

**Xianxi Wu:** investigation, validation. **Guanglei Zhang:** data curation, formal analysis, methodology, investigation, visualization, writing – original draft. **Gaocheng Shi:** formal analysis, validation. **Mengqi Zhao:** data curation, validation. **Xiaoyong Yuan:** funding acquisition. **Cong Wang:** software, data curation, methodology, visualization. **Zongmeng Zhang:** validation, writing – review and editing. **Hao Yu:** funding acquisition, methodology, project administration, supervision, writing – review and editing.

## Funding

This study was supported by Drug Administration of Anhui Province Top Ten Medicine of Anhui (Chuzhou Chrysanthemum) Demonstration Base Project (880175); Development and Research of Compound Dendrobium Instant Powder for Blood Sugar Reduction (BYH202314); Research and Development Project of Big Health Products (BYH2026036); Anhui Provincial Higher Education Institutions Natural Science Research Project (2024AH051306, 2025AHGXZK40776); Bozhou City Science and Technology Plan Project (bzzd2024007).

## Disclosure

No generative artificial intelligence tools were adopted for data analysis, manuscript writing, figure drawing, or any other procedures in the current study.

## Ethics Statement

All animal housing and experimental procedures were approved by the Animal Ethics Committee of Anhui Science and Technology University (Chuzhou, China; approval number: AK2024044) and were carried out in compliance with the ARRIVE 2.0 guidelines.

## Conflicts of Interest

The authors declare no conflicts of interest.

## Supporting information


**Table S1:** Grading scale for the extent of neurological injury in rats.
**Table S2:** Gradient elution program.
**Table S3:** Detailed Information and Changes in Differential Metabolites.

## Data Availability

The data that support the findings of this study are available from the corresponding author upon reasonable request.

## References

[fsn372216-bib-0001] Amani, H. , E. Mostafavi , M. R. Alebouyeh , et al. 2019. “Would Colloidal Gold Nanocarriers Present an Effective Diagnosis or Treatment for Ischemic Stroke?” International Journal of Nanomedicine 14: 8013–8031. 10.2147/IJN.S210035. 31632015 PMC6789974

[fsn372216-bib-0002] Andersen, K. K. , T. S. Olsen , C. Dehlendorff , and L. P. Kammersgaard . 2009. “Hemorrhagic and Ischemic Strokes Compared: Stroke Severity, Mortality, and Risk Factors.” Stroke 40, no. 6: 2068–2072. 10.1161/STROKEAHA.108.540112. 19359645

[fsn372216-bib-0003] Bartman, C. R. , D. R. Weilandt , Y. Shen , et al. 2023. “Slow TCA Flux and ATP Production in Primary Solid Tumours but Not Metastases.” Nature 614, no. 7947: 349–357. 10.1038/s41586-022-05661-6. 36725930 PMC10288502

[fsn372216-bib-0004] Cao, L. , D. Zhang , J. Chen , et al. 2017. “G6PD Plays a Neuroprotective Role in Brain Ischemia Through Promoting Pentose Phosphate Pathway.” Free Radical Biology & Medicine 112: 433–444. 10.1016/j.freeradbiomed.2017.08.011. 28823591

[fsn372216-bib-0005] Chandel, N. S. 2021. “NADPH‐The Forgotten Reducing Equivalent.” Cold Spring Harbor Perspectives in Biology 13, no. 6: a040550. 10.1101/cshperspect.a040550. 34074675 PMC8168431

[fsn372216-bib-0006] Chen, L. , Y. Chao , P. Cheng , N. Li , H. Zheng , and Y. Yang . 2019. “UPLC‐QTOF/MS‐Based Metabolomics Reveals the Protective Mechanism of Hydrogen on Mice With Ischemic Stroke.” Neurochemical Research 44, no. 8: 1950–1963. 10.1007/s11064-019-02829-x. 31236794

[fsn372216-bib-0007] Chi, O. Z. , X. Liu , S. Cofano , N. Patel , E. Jacinto , and H. R. Weiss . 2021. “Rapalink‐1 Increased Infarct Size in Early Cerebral Ischemia‐Reperfusion With Increased Blood‐Brain Barrier Disruption.” Frontiers in Physiology 12: 706528. 10.3389/fphys.2021.706528. 34354602 PMC8329705

[fsn372216-bib-0008] Gerschenfeld, G. , I. P. Muresan , R. Blanc , et al. 2017. “Two Paradigms for Endovascular Thrombectomy After Intravenous Thrombolysis for Acute Ischemic Stroke.” JAMA Neurology 74, no. 5: 549–556. 10.1001/jamaneurol.2016.5823. 28319240 PMC5822198

[fsn372216-bib-0009] Han, Y. , M. Zhou , L. Wang , et al. 2015. “Comparative Evaluation of Different Cultivars of Flos Chrysanthemi by an Anti‐Inflammatory‐Based NF‐kappaB Reporter Gene Assay Coupled to UPLC‐Q/TOF MS With PCA and ANN.” Journal of Ethnopharmacology 174: 387–395. 10.1016/j.jep.2015.08.044. 26320691

[fsn372216-bib-0010] Hasseldam, H. , R. S. Rasmussen , H. H. El Ali , and F. F. Johansen . 2024. “N‐Acetyl Aspartate Levels Early After Ischemic Stroke Accurately Reflect Long‐Term Brain Damage.” Heliyon 10, no. 2: e24233. 10.1016/j.heliyon.2024.e24233. 38293500 PMC10825333

[fsn372216-bib-0011] Hu, X. , T. M. De Silva , J. Chen , and F. M. Faraci . 2017. “Cerebral Vascular Disease and Neurovascular Injury in Ischemic Stroke.” Circulation Research 120, no. 3: 449–471. 10.1161/CIRCRESAHA.116.308427. 28154097 PMC5313039

[fsn372216-bib-0012] Huang, Z. , L. Guo , L. Huang , Y. Shi , J. Liang , and L. Zhao . 2021. “Baicalin‐Loaded Macrophage‐Derived Exosomes Ameliorate Ischemic Brain Injury via the Antioxidative Pathway.” Materials Science & Engineering, C: Materials for Biological Applications 126: 112123. 10.1016/j.msec.2021.112123. 34082940

[fsn372216-bib-0013] Joya, A. , M. Ardaya , A. Montilla , et al. 2021. “In Vivo Multimodal Imaging of Adenosine A(1) Receptors in Neuroinflammation After Experimental Stroke.” Theranostics 11, no. 1: 410–425. 10.7150/thno.51046. 33391483 PMC7681082

[fsn372216-bib-0014] Kaplan‐Arabaci, O. , A. Acari , P. Ciftci , and D. Gozuacik . 2022. “Glutamate Scavenging as a Neuroreparative Strategy in Ischemic Stroke.” Frontiers in Pharmacology 13: 866738. 10.3389/fphar.2022.866738. 35401202 PMC8984161

[fsn372216-bib-0015] Kierans, S. J. , and C. T. Taylor . 2021. “Regulation of Glycolysis by the Hypoxia‐Inducible Factor (HIF): Implications for Cellular Physiology.” Journal of Physiology 599, no. 1: 23–37. 10.1113/JP280572. 33006160

[fsn372216-bib-0016] Krzyzanowska, W. , B. Pomierny , B. Bystrowska , et al. 2017. “Ceftriaxone‐ and N‐Acetylcysteine‐Induced Brain Tolerance to Ischemia: Influence on Glutamate Levels in Focal Cerebral Ischemia.” PLoS One 12, no. 10: e0186243. 10.1371/journal.pone.0186243. 29045497 PMC5646803

[fsn372216-bib-0017] Lei, Y. , X. Jin , M. Sun , and Z. Ji . 2021. “miR‐129‐5p Ameliorates Ischemic Brain Injury by Binding to SIAH1 and Activating the mTOR Signaling Pathway.” Journal of Molecular Neuroscience 71, no. 9: 1761–1771. 10.1007/s12031-021-01872-0. 34355355

[fsn372216-bib-0018] Li, M. , Z. P. Zhou , M. Sun , et al. 2016. “Reduced Nicotinamide Adenine Dinucleotide Phosphate, a Pentose Phosphate Pathway Product, Might be a Novel Drug Candidate for Ischemic Stroke.” Stroke 47, no. 1: 187–195. 10.1161/STROKEAHA.115.009687. 26564104

[fsn372216-bib-0019] Lim, H. S. , O. S. Kim , B. Y. Kim , and S. J. Jeong . 2016. “Apigetrin From Scutellaria Baicalensis Georgi Inhibits Neuroinflammation in BV‐2 Microglia and Exerts Neuroprotective Effect in HT22 Hippocampal Cells.” Journal of Medicinal Food 19, no. 11: 1032–1040. 10.1089/jmf.2016.0074. 27845861

[fsn372216-bib-0020] Lo, B. W. Y. , and H. Fukuda . 2025. “Advances in Ischemic Stroke Treatment: Current and Future Therapies.” Neurology and Therapy 14, no. 5: 1783–1796. 10.1007/s40120-025-00810-1. 40797003 PMC12450147

[fsn372216-bib-0021] Luo, S. , H. Li , Z. Mo , et al. 2019. “Connectivity Map Identifies Luteolin as a Treatment Option of Ischemic Stroke by Inhibiting MMP9 and Activation of the PI3K/Akt Signaling Pathway.” Experimental and Molecular Medicine 51, no. 3: 1–11. 10.1038/s12276-019-0229-z. PMC6434019 30911000

[fsn372216-bib-0022] Ma, Q. , R. Li , L. Wang , et al. 2021. “Temporal Trend and Attributable Risk Factors of Stroke Burden in China, 1990‐2019: An Analysis for the Global Burden of Disease Study 2019.” Lancet Public Health 6, no. 12: e897–e906. 10.1016/S2468-2667(21)00228-0. 34838196 PMC9047702

[fsn372216-bib-0023] Pan, Y. W. , D. P. Wu , H. F. Liang , et al. 2022. “Total Saponins of Panax Notoginseng Activate Akt/mTOR Pathway and Exhibit Neuroprotection In Vitro and In Vivo Against Ischemic Damage.” Chinese Journal of Integrative Medicine 28, no. 5: 410–418. 10.1007/s11655-021-3454-y. 34581940

[fsn372216-bib-0024] Powers, W. J. 2020. “Acute Ischemic Stroke.” New England Journal of Medicine 383, no. 3: 252–260. 10.1056/NEJMcp1917030. 32668115

[fsn372216-bib-0025] Puig, B. , S. Brenna , and T. Magnus . 2018. “Molecular Communication of a Dying Neuron in Stroke.” International Journal of Molecular Sciences 19, no. 9: 2834. 10.3390/ijms19092834. 30235837 PMC6164443

[fsn372216-bib-0026] Szwed, A. , E. Kim , and E. Jacinto . 2021. “Regulation and Metabolic Functions of mTORC1 and mTORC2.” Physiological Reviews 101, no. 3: 1371–1426. 10.1152/physrev.00026.2020. 33599151 PMC8424549

[fsn372216-bib-0027] Vaskova, J. , L. Kocan , L. Vasko , and P. Perjesi . 2023. “Glutathione‐Related Enzymes and Proteins: A Review.” Molecules 28, no. 3: 1447. 10.3390/molecules28031447. 36771108 PMC9919958

[fsn372216-bib-0028] Villa‐Gonzalez, M. , G. Martin‐Lopez , and M. J. Perez‐Alvarez . 2022. “Dysregulation of mTOR Signaling After Brain Ischemia.” International Journal of Molecular Sciences 23, no. 5: 2814. 10.3390/ijms23052814. 35269956 PMC8911477

[fsn372216-bib-0029] Wang, C. , H. Chen , H. H. Jiang , B. B. Mao , and H. Yu . 2021. “Total Flavonoids of Chuju Decrease Oxidative Stress and Cell Apoptosis in Ischemic Stroke Rats: Network and Experimental Analyses.” Frontiers in Neuroscience 15: 772401. 10.3389/fnins.2021.772401. 34955724 PMC8695723

[fsn372216-bib-0030] Wang, C. , Z. Ma , Z. Wang , et al. 2021. “Eriodictyol Attenuates MCAO‐Induced Brain Injury and Neurological Deficits via Reversing the Autophagy Dysfunction.” Frontiers in Systems Neuroscience 15: 655125. 10.3389/fnsys.2021.655125. 34122022 PMC8190663

[fsn372216-bib-0031] Wang, F. X. , G. Mu , Z. H. Yu , et al. 2025. “Lactylation: A Promising Therapeutic Target in Ischemia‐Reperfusion Injury Management.” Cell Death Discovery 11, no. 1: 100. 10.1038/s41420-025-02381-4. 40082399 PMC11906755

[fsn372216-bib-0032] Wang, Y. , L. Zhou , N. Wang , et al. 2025. “Comprehensive Characterization of Metabolic Consumption and Production by the Human Brain.” Neuron 113, no. 11: 1708–1722. 10.1016/j.neuron.2025.03.003. 40147438

[fsn372216-bib-0033] Wu, S. , X. Piao , N. Wang , and Y. Zhai . 2020. “Naoluo Xintong Capsule Ameliorates Apoptosis Induced by Endoplasmic Reticulum Stress in Rats With Cerebral Ischemia/ Reperfusion Injury.” Annals of Palliative Medicine 9, no. 5: 2913–2925. 10.21037/apm-20-387. 32787375

[fsn372216-bib-0034] Yang, M. , B. Liu , B. Chen , Y. Shen , and G. Liu . 2025. “Cerebral Ischemia‐Reperfusion Injury: Mechanisms and Promising Therapies.” Frontiers in Pharmacology 16: 1613464. 10.3389/fphar.2025.1613464. 40766753 PMC12323178

[fsn372216-bib-0035] Yin, Y. , W. Nie , Z. Q. Tang , and S. J. Zhu . 2025. “Flavonoid‐Rich Extracts From Chuju (Asteraceae Chrysanthemum L.) Alleviate the Disturbance of Glycolipid Metabolism on Type 2 Diabetic Mice via Modulating the Gut Microbiota.” Food 14, no. 5: 765. 10.3390/foods14050765. PMC11898795 40077469

[fsn372216-bib-0036] Zhan, G. , M. Long , K. Shan , C. Xie , and R. Yang . 2022. “Antioxidant Effect of *Chrysanthemum morifolium* (Chuju) Extract on H(2)O(2)‐Treated L‐O2 Cells as Revealed by LC/MS‐Based Metabolic Profiling.” Antioxidants (Basel) 11, no. 6: 1068. 10.3390/antiox11061068. 35739965 PMC9219928

[fsn372216-bib-0037] Zhang, Q. , M. Jia , Y. Wang , Q. Wang , and J. Wu . 2022. “Cell Death Mechanisms in Cerebral Ischemia‐Reperfusion Injury.” Neurochemical Research 47, no. 12: 3525–3542. 10.1007/s11064-022-03697-8. 35976487

[fsn372216-bib-0038] Zhang, Z. , J. Chen , F. Chen , et al. 2018. “Tauroursodeoxycholic Acid Alleviates Secondary Injury in the Spinal Cord via Up‐Regulation of CIBZ Gene.” Cell Stress & Chaperones 23, no. 4: 551–560. 10.1007/s12192-017-0862-1. 29151236 PMC6045539

