# Supplementary material for: Investigation of the Mechanism of Total Flavonoids From Chuju in Mitigating Cerebral Ischemia–Reperfusion Injury in Rats Using Untargeted Metabolomics
Source: Food Sci Nutr. 2026 Aug 27;14(9):e72216. doi: 10.1002/fsn3.72216 (PMC13522845; doi:10.1002/fsn3.72216)
Supplement: Supplementary file 1 [file FSN3-14-e72216-s001.docx]

**Supplementary Table 1** Grading scale for the extent of neurological injury in rats.

| Grade | Degree of Neurological Injury |
| --- | --- |
| Ⅰ | No signs of neurological injury |
| Ⅱ | Inability to fully extend the left forepaw |
| Ⅲ | Inability to fully extend the left forepaw and Circling to the left during ambulation. |
| Ⅳ | The inability to fully extend the left forepaw. When walking, there is a tendency toward hemiplegia on the left side. |
| Ⅴ | Loss of consciousness, unconsciousness, and an inability to walk spontaneously. |

**Supplementary Table 2** Gradient elution program.

| Time (min) | Component gradient ratio (B%) |
| --- | --- |
| 0 | 1 |
| 1 | 1 |
| 10 | 99 |
| 13 | 99 |
| 14 | 1 |
| 17 | 1 |

**Supplementary Table 3** Detailed Information and Changes in Differential Metabolites

| Number | Name | MF | HMDBID | P value | VIP | Model/Sham | TFCJ/Model | Metabolic pathways |
| --- | --- | --- | --- | --- | --- | --- | --- | --- |
| 1 | succinic acid | C_4_H_6_O_4_ | HMDB0000254 | 0.000638 | 1.0239 | ↓ | ↑ | Tricarboxylic acid cycle（TCA Cycle） |
| 2 | 2-oxoglutarate | C_5_H_6_O_5_ | HMDB0000208 | 0.000978 | 1.2805 | ↓ | ↑ | Tricarboxylic acid cycle（TCA Cycle） |
| 3 | fumaric acid | C_4_H_4_O_4_ | HMDB0000134 | 0.043583 | 1.4943 | ↓ | ↑ | Tricarboxylic acid cycle（TCA Cycle） |
| 4 | citric acid | C_6_H_8_O_7_ | HMDB0000094 | 0.000000 | 1.3308 | ↓ | ↑ | Tricarboxylic acid cycle（TCA Cycle） |
| 5 | lactic acid | C_3_H_6_O_3_ | HMDB0000190 | 0.013824 | 1.1715 | ↑ | ↓ | Glycoilsis |
| 6 | phosphoenolpyruvate | C_3_H_5_O_6_P | HMDB0000263 | 0.012296 | 1.0579 | ↑ | ↓ | Glycolysis |
| 7 | D-fructofuranose 6-phosphate | C_6_H_13_O_9_P | HMDB0003971 | 0.001162 | 1.1355 | ↑ | ↑ | Glycolysis |
| 8 | 1-(beta-D-ribofuranosyl)-1,4-dihydronicotinamide | C_11_H_16_N_2_O_5_ | HMDB0000855 | 0.030503 | 1.1140 | ↓ | ↑ | Nicotinate and Nicotinamide Metabolism |
| 9 | 6-phospho-D-gluconic acid | C_6_H_13_O_10_P | HMDB0001316 | 0.000122 | 1.2116 | ↑ | ↑ | Pentose Phosphate Pathway |
| 10 | D-glucopyranose 6-phosphate | C_6_H_13_O_9_P | HMDB0001401 | 0.004074 | 2.0823 | ↑ | ↑ | Pentose Phosphate Pathway, Glycolysis |
| 11 | D-glucono-1,5-lactone | C_6_H_10_O_6_ | HMDB0000150 | 0.030664 | 1.0240 | ↑ | ↑ | Pentose Phosphate Pathway |
| 12 | L-Glutamine | C_5_H_10_N_2_O_3_ | HMDB0000641 | 0.021534 | 1.2295 | ↓ | ↑ | Aspartate and glutamate metabolis |
| 13 | N-acetyl-L-aspartate | C_6_H_9_NO_5_ | HMDB0000812 | 0.000000 | 1.3150 | ↓ | ↑ | Aspartate and glutamate metabolis |
| 14 | L-glutamic acid | C_5_H_9_NO_4_ | HMDB0000148 | 0.000139 | 1.2373 | ↑ | ↓ | Aspartate and glutamate metabolis |
| 15 | glutathione | C_10_H_17_N_3_O_6_S | HMDB0000125 | 0.000108 | 1.5042 | ↓ | ↑ | Aspartate and glutamate metabolis |
| 16 | adenosine 5'-monophosphate | C_10_H_14_N_5_O_7_P | HMDB0000045 | 0.008100 | 1.1378 | ↓ | ↑ | Aspartate and glutamate metabolis |
| 17 | hypoxanthine | C_5_H_4_N_4_O | HMDB0000157 | 0.003774 | 1.0389 | ↓ | ↑ | Purine Metabolism |
| 18 | xanthine | C_5_H_4_N_4_O_2_ | HMDB0000292 | 0.019128 | 1.1270 | ↑ | ↓ | Purine Metabolism |
| 19 | adenosine | C_10_H_13_N_5_O_4_ | HMDB0000050 | 0.026007 | 1.1970 | ↓ | ↑ | Nucleotide metabolism |
| 20 | octanoic acid | C_8_H_16_O_2_ | HMDB0000482 | 0.032163 | 1.1775 | ↓ | ↑ | Fatty Acid Metabolism |
| 21 | quinolinic acid | C_7_H_5_NO_4_ | HMDB0000232 | 0.000000 | 1.0424 | ↑ | ↓ | NAD Biosynthesis |
| 22 | 12-hydroxylauric acid | C_12_H_24_O_3_ | HMDB0002059 | 0.000672 | 1.0660 | ↑ | ↓ | Cell signaling |
| 23 | L-asparagine | C_4_H_8_N_2_O_3_ | HMDB0000168 | 0.001745 | 1.1223 | ↓ | ↑ | Aspartate and glutamate metabolism |
| 24 | alpha-tocopherol | C_29_H_50_O_2_ | HMDB0001893 | 0.000006 | 1.1911 | ↑ | ↓ | Biosynthesis of secondary metabolites |
| 25 | pyruvate | C_3_H_4_O_3_ | HMDB0000243 | 0.001774 | 1.3844 | ↓ | ↑ | Glycolysis |
